# Mental health status of secondary school students: a meta-analysis of comparative studies between one-child and multi-child families in China

**DOI:** 10.3389/fpsyt.2025.1594968

**Published:** 2025-08-07

**Authors:** Wei Zhang, Pan Chen, Shu-Ying Rao, Yuan-Yuan Jiang, Zhaohui Su, Teris Cheung, Chee H. Ng, Yu-Tao Xiang, Gang Wang

**Affiliations:** ^1^ Unit of Psychiatry, Department of Public Health and Medicinal Administration, & Institute of Translational Medicine, Faculty of Health Sciences, University of Macau, Macao, Macao SAR, China; ^2^ Centre for Cognitive and Brain Sciences, University of Macau, Macao, Macao SAR, China; ^3^ School of Public Health, Southeast University, Nanjing, China; ^4^ School of Nursing, Hong Kong Polytechnic University, Hong Kong, Hong Kong SAR, China; ^5^ Department of Psychiatry, The Melbourne Clinic and St Vincent’s Hospital, University of Melbourne, Richmond, VIC, Australia; ^6^ Beijing Key Laboratory of Mental Disorders, National Clinical Research Center for Mental Disorders & National Center for Mental Disorders, Beijing Anding Hospital, Capital Medical University, Beijing, China

**Keywords:** mental health, meta-analysis, multi-child families, one-child families, secondary school students

## Abstract

**Introduction:**

Mental health problems are common among secondary school students. However, when comparing one-child and multi-child families, the findings on the mental health of students are mixed. Therefore, we conducted a meta-analysis to compare the mental health status between secondary school students from one-child and multi-child families in China.

**Methods:**

Relevant studies using standard instruments on mental health (e.g., the Middle School Student Mental Health Scale; MSSMHS and the Mental Health Test; MHT) were searched in PubMed, Web of Science, PsycINFO, China National Knowledge Infrastructure (CNKI), and Wanfang. A random-effects model was employed to compute the pooled effect size. Subgroup analyses for categorical variables and meta-regression analyses for continuous variables were carried out to examine the potential moderators of group differences.

**Results:**

We identified 39 studies, which included 11,889 secondary school students from one-child families and 13,795 from multi-child families. No significant difference in mental health was found between students from one-child and multi-child families. However, significant group differences were observed in certain MHT domains, including Learning Anxiety [95% confidence interval (CI): -0.19; 0.00, I² = 0.0%, P = 0.04], Social Anxiety (95% CI:-0.25; 0.00, I² = 45.8%, P = 0.04), Tendency Towards Self-Blame (95% CI: -0.23; -0.07, I² = 0.0%, P < 0.01) and Allergic Tendencies (95% CI: -0.25; -0.01, I² =43.5%, P = 0.04).

**Discussion:**

This meta-analysis did not show significant differences in the mental health between students from one-child and multi-child families. Future research should investigate the influence of socio-demographic factors, such as gender and place of residence, on the mental health of this population.

**Systematic review registration:**

https://inplasy.com/wp-content/uploads/2024/03/INPLASY-Protocol-5996.pdf, identifier INPLASY202430053.

## 1 Introduction

During a stage of rapid physical and psychological development, secondary school students often encounter mental health problems, such as anxiety and depression, which can significantly affect their academic performance and quality of life (1). High rates of mental health problems among secondary school students have been reported, for instance, 27% experienced anxiety, 24% suffered from depression, 17% developed sleep disorders, 22% engaged in self-harm behaviors, 17% had suicidal intentions and 7% made suicide plans (2). Common stressors that may increase their susceptibility to mental health problems include peer and family conflicts, social anxiety, and body image concerns (3–5). Further, secondary school students in China often confront considerable academic pressures in preparing for their ‘Zhongkao’ (Senior High School Entrance Examination), ‘Gaokao’ (National College Entrance Examination) and future career (6). Thus, research focusing on the mental health of secondary school students is crucial to address this growing challenge.

According to the Family Systems Theory (7), the behaviours of family members are usually shaped by family structure and interconnection. The Resource Dilution Theory suggests that as the number of children within the family increases, the resources for each child reduces, leading to more competition and conflict (8). In only-child families, children typically have good emotional and material support from their parents, resulting in strong parent-child relationships (9, 10). In families with siblings, children tend to compete for parental attention often resulting in sibling rivalry. According to previous research, up to 50% of children experience sibling bullying (11, 12). Although moderate sibling rivalry may help enhance social and cognitive skills (13), it can also worsen behavioral and emotional issues when it escalates to bullying.

The implementation of China’s stringent one-child policy has had profound impact on the country’s social structure and family dynamics. Initiated in the early 1980s, the one-child policy was a population control measure aimed at reducing population growth and alleviating pressures on societal resources (14). This policy restricted most families to having a single child, with some exceptions such as families from ethnic minorities or those with severely disabled children (15). However, in response to changes in socioeconomic development, the Chinese government officially changed the one-child policy at the end of 2015, allowing each family to have two children (16). This major policy shift might have considerable implications for the mental health and well-being of Chinese adolescents, which warrants in-depth research investigation.

Several validated measurement tools have been routinely employed in the assessment of mental health of children and adolescents, such as the Symptom Checklist-90 (SCL-90) that has been previously used to evaluate the mental health of secondary school students in one-child and multi-child families (17). However, as SCL-90 targets both adults and adolescents (18), it may not be sensitive enough to detect small changes in the mental health of secondary school students. Instead, a number of standardized measures have been developed explicitly for middle school students, including the Middle School Student Mental Health Scale (MSSMHS) (19) and the Mental Health Test (MHT) (20). Compared to the commonly used SCL-90 (21), MSSMHS and MHT are more specific in addressing psychological issues related to secondary school students such as academic stress and peer relationships (19, 20). In contrast, the SCL-90 is designed for a broader population (22). Additionally, MSSMHS and MHT have been validated in multiple local studies, demonstrating high reliability and validity. The test-retest reliability of MSSMHS ranged from 0.716 to 0.905 (23), and the Cronbach’s α coefficient of MHT was greater than 0.85 (24, 25), indicating good psychometric properties in measuring the mental health of secondary school students.

Previous studies on the mental health of secondary school students from one-child families in China were mostly cross-section in design and different in terms of sampling methods, selection criteria, and sample sizes (26). As a result, the findings comparing mental health status of secondary school students between one-child and multi-child families have been mixed (26, 27). Previous meta-analyses have primarily focused on the overall rates of mental health problems among secondary school students (2) or those across all age groups from both one-child and multi-child families (26).

To fill this gap, we undertook a meta-analysis to compare the mental health status of secondary school students between one-child and multi-child families in China, as well as explored their potential moderating factors.

## 2 Materials and methods

### 2.1 Search strategy

This systematic review and meta-analysis, registered with the number INPLASY202430053, included relevant studies published until August 28, 2023. Four researchers (WZ, PC, YYJ, and SYR) conducted an independent literature search in PubMed, Web of Science, PsycINFO, China National Knowledge Infrastructure (CNKI), and Wanfang. The detailed search strategy and search terms are listed in **Supplementary Table S1**.

### 2.2 Inclusion and exclusion criteria

**Figure 1:**
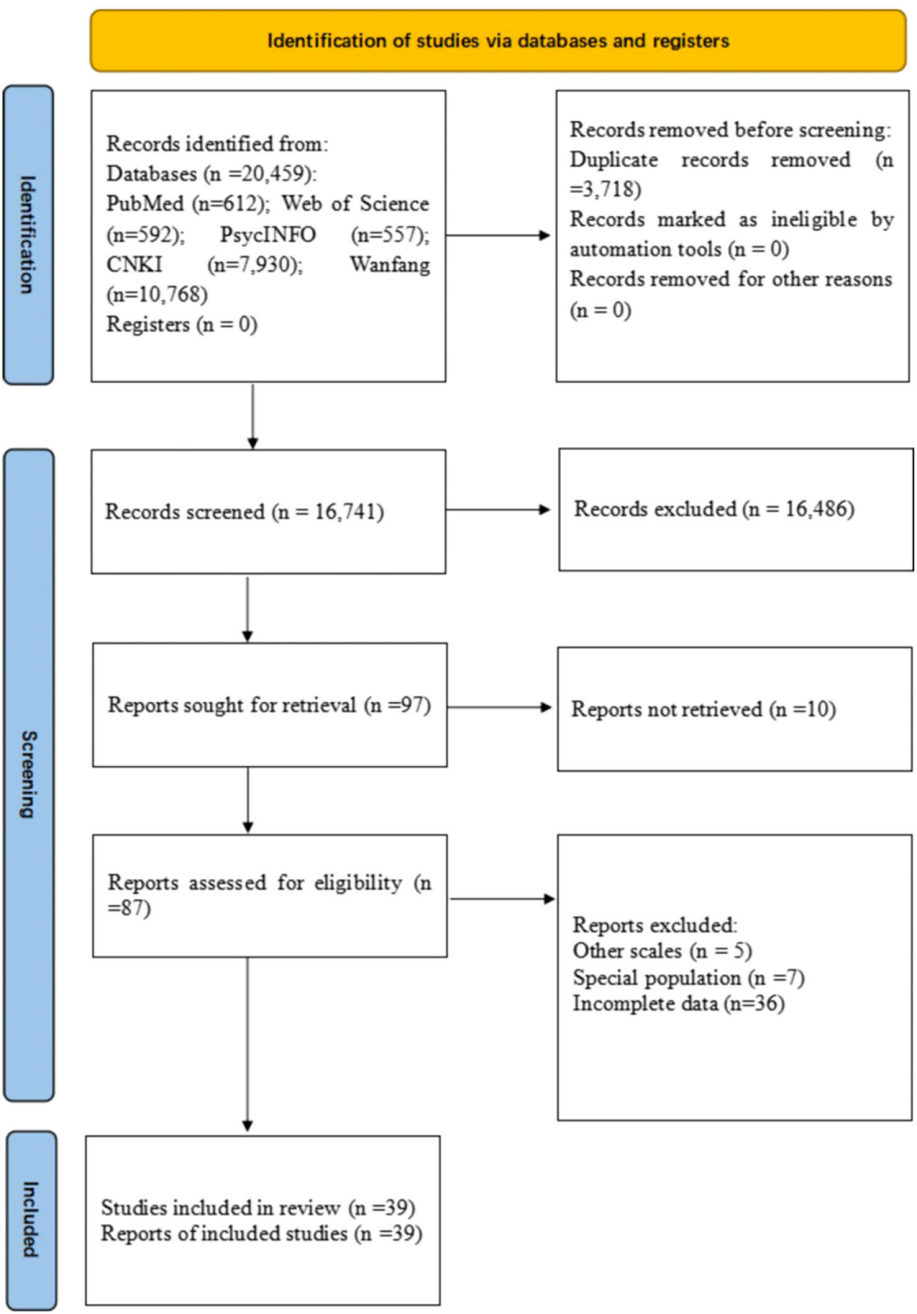
Flow diagram of study selection procedure.

To be eligible, participants were secondary school students from one-child families, and controls were secondary school students from multi-child households. Additionally, mental health status was measured with standardized scales specifically created for middle school students like the MHT (20) and MSSMHS (19). Cross-sectional comparative studies were adopted. Exclusion criteria included studies involving specific groups, such as ethnic minorities, and those conducted during the COVID-19 pandemic. As previously recommended (21, 28), studies conducted in specific groups and certain periods were excluded, such as, family members spending extended periods at home or during the COVID-19 pandemic, since this might increase parental stress or family conflict (29, 30). Such factors could distort the impact of family structure on children’s mental health. The four researchers independently screened the literature by reviewing titles and abstracts, and subsequently examined the full texts. Any inconsistencies encountered during the literature selection phase were addressed through consultations with the senior researcher (YTX). The literature methodology is introduced in **Figure 1**.

### 2.3 Data extraction and study quality assessment

The data extraction of study (e.g., study title, author details, publication year, timing and location of survey, type of instruments, study design and sampling methods) and participant characteristics (e.g., mean age and type of families such as one-child and multi-child families) were performed independently by the same four researchers (**Table 1**).

**Table 1:** Characteristics of studies included in this meta-analysis.

No.	First author and publication year	Sample size (N=25,684)			Standardized scale total (mean) score				Region	Sampling method	Population	Age (mean ± SD; years)	Scales	Quality assessment score
		Total	Only child (n=11,889)	Non-only child (n=13,795)	Only child (Mean or total)	Only child (SD)	Non-only child (Mean or total)	Non-only child (SD)						
1	Cai (2018) (31)	479	93	386	2.11	0.71	2.12	0.65	Hebei	NR	Senior High	NR	MSSMHS	6
2	Chen (2014) (32)	822	132	690	2.61	0.70	2.24	0.58	Hebei	Cluster, Random	Both	NR	MSSMHS	6
3	Cheng (2006) (33)	956	680	276	1.94	0.56	2.01	0.55	Henan	Cluster	Senior High	16.6 ± 1.1	MSSMHS	6
4	Feng (2013) (34)	384	319	65	127.02	38.87	137.47	38.32	Tianjin	Random	Junior High	NR	MSSMHS	5
5	Ge (2012) (35)	469	180	276	2.21	0.62	2.22	0.59	Jiangsu	Random	Junior High	NR	MSSMHS	6
6	Guo (2019) (36)	757	349	408	1.92	0.63	1.96	0.62	Sichuan	Purposeful	Junior High	NR	MSSMHS	5
7	Han (2022) (37)	1,104	235	869	1.42	0.57	1.46	0.63	Hebei	Random	Junior High	NR	MSSMHS	5
8	Huang (2017) (38)	219	46	173	2.15	0.71	2.08	0.67	Guangdong	Random	Senior High	NR	MSSMHS	7
9	Li (2001) (39)	196	172	24	36.81	9.66	36.29	9.54	Chongqing	Random	Junior High	NR	MHT	4
10	Li (2017) (40)	1,709	1,006	703	1.87	0.63	1.87	0.57	NA	Stratified	Junior High	NR	MSSMHS	6
11	Li (2021) (41)	446	87	358	2.26	0.64	2.30	0.67	Hubei	NR	Senior High	14.86 ± 0.52	MSSMHS	5
12	Liu (2012) (42)	393	271	122	2.16	0.57	2.11	0.57	Chongqing	Random	Both	NR	MSSMHS	6
13	Liu (2017a) (43)	392	313	79	104.65	30.14	111.39	38.09	Heilongjiang	NR	Junior High	NR	MSSMHS	5
14	Liu (2017b) (44)	825	262	563	2.31	0.57	2.31	0.54	Hunan	NR	Both	NR	MSSMHS	5
15	Liu (2020) (45)	536	93	443	2.28	0.76	1.97	0.68	Guangxi	Random	Junior High	NR	MSSMHS	6
16	Liu (2011) (46)	1,918	1,304	614	1.96	0.54	2.05	0.59	Anhui	Stratified, Cluster Random	Junior High	NR	MSSMHS	6
17	Lu (2019) (47)	904	508	395	37.66	15.92	42.52	13.98	Shandong	Cluster	Senior High	NR	MHT	6
18	Luo (2017) (48)	522	129	343	42.30	12.10	46.00	13.60	Guangdong	NR	Junior High	NR	MHT	6
19	Ma (2017) (49)	385	135	250	124.09	41.93	129.09	35.36	Hebei	Cluster	Junior High	NR	MSSMHS	6
20	Peng (2017) (50)	702	116	586	36.30	14.07	36.94	14.06	Guangdong	NR	Junior High	NR	MHT	6
21	Qiao (2016) (51)	285	148	137	106.35	32.70	102.21	30.87	Xinjiang	Stratified, Cluster, Random	Junior High	NR	MSSMHS	6
22	Qin (2019) (52)	921	113	810	2.40	0.64	2.29	0.59	Guangxi	Stratified, Cluster	Junior High	NR	MSSMHS	6
23	Shi (2010) (53)	335	84	251	2.13	0.57	2.16	0.56	Hebei	Cluster, Random	Senior High	17.28 ± 0.954	MSSMHS	6
24	Sun (2016) (54)	476	111	365	2.17	0.67	2.01	0.66	Hebei	Random	Senior High	NR	MSSMHS	6
25	Tang (2015) (55)	611	465	146	37.18	15.32	37.49	12.72	Shanghai	Random	Junior High	NR	MHT	5
26	Tang (2010) (56)	809	567	242	1.85	0.51	1.86	0.54	Jiangsu	Cluster, Random	Junior High	NR	MSSMHS	6
27	Tian (2011) (57)	1,863	1,084	779	1.88	0.55	1.92	0.50	Liaoning	Cluster, Random	Senior High	16.23 ± 0.64	MSSMHS	6
28	Wang (2018) (58)	860	445	415	2.11	0.60	2.09	0.55	Shandong	NR	Senior High	NR	MSSMHS	6
29	Wang (2022) (59)	497	284	213	1.91	0.75	1.97	0.72	Qinghai	Stratified, Random	Junior High	13.36	MSSMHS	6
30	Wang (2021) (60)	159	61	98	2.08	0.79	2.14	0.82	Henan	NR	Junior High	14.86 ± 0.533	MSSMHS	5
31	Xiang (2021) (61)	352	223	129	111.93	31.36	115.22	27.75	Xinjiang	Convenient	Both	NR	MSSMHS	6
32	Xiao (2016) (62)	654	170	461	2.07	0.46	2.15	0.52	Hubei	Cluster, Random	Senior High	NR	MSSMHS	6
33	Xie (2020) (63)	558	220	348	2.34	0.61	2.31	0.61	Hebei	NR	Senior High	NR	MSSMHS	6
34	Zhang (2012) (64)	441	215	226	1.62	0.45	1.78	0.55	Shandong	NR	Junior High	NR	MSSMHS	5
35	Zhang (2021) (65)	543	122	421	99.20	37.20	101.12	41.01	Shandong	NR	Junior High	NR	MSSMHS	4
36	Zhao (2011) (66)	285	24	261	34.58	12.28	33.68	11.39	Henan	Random	Senior High	NR	MHT	6
37	Zhao (2014) (67)	502	331	171	1.81	0.53	1.97	0.58	Jiangsu	Random	Junior High	NR	MSSMHS	6
38	Zhao (2022) (68)	395	253	142	1.85	0.59	2.00	0.64	Heilongjiang	Random	Senior High	NR	MSSMHS	5
39	Zhou (2014) (69)	1,096	539	557	2.02	0.56	2.02	0.52	Zhejiang	Stratified, Cluster, Random	Both	NR	MSSMHS	6

MHT, Mental health test; MSSMHS, Middle School Student Mental Health Scale; NR, Not reported; SD, Standard deviation.

Study quality was independently assessed by the four researchers utilizing an 8-point instrument designed for epidemiological studies (70, 71) (**Supplementary Table S1**). Each of the items in the appraisal tool scored one point. The studies could be classified as low (0–3 points), moderate (4–6 points), or high (7–8 points) quality according to the total score (72). The agreement between researchers was above 0.8. In cases of discrepancies, consensus was reached through discussion, and any issues were resolved through consultation with another researcher (YTX).

### 2.4 Statistical analysis

Data were analyzed utilizing R software (version 4.3.2) (73). A random-effects model was utilized to calculate the combined effect size, specifically the standard mean difference (SMD), along with 95% confidence intervals (CIs) for each study. The heterogeneity among studies was evaluated using the I² statistic. An I² value exceeding 50% indicated substantial heterogeneity. Subgroup analyses for categorical variables and meta-regression analyses for continuous variables were conducted to explore potential moderators of group difference. A funnel plot and Egger’s test were employed to assess publication bias. Additionally, a sensitivity analysis was conducted to ascertain the robustness and reliability of the primary results by removing studies one by one. Significance level was set at 0.05 (two-tailed test).

## 3 Results

### 3.1 Study characteristics

In total, 20,459 relevant publications were identified. After the removal of 3,718 duplicates, 16,417 titles and abstracts were screened, and the full text of 97 papers were assessed for eligibility (**Figure 1**). Finally, 39 studies from across 18 provinces or municipalities in China, with 11,889 participants from one-child families and 13,795 from multi-child families were included. The mean age of the participants ranged from 13.36 to 17.28 years. Geographically, most studies were conducted in eastern China (48.7%, n = 19), followed by central (20.5%, n = 8), western China (20.5%, n = 8), and northeast China (7.7%, n = 3). All studies were cross-sectional, with 61.5% (n = 24) employing probability sampling methods.

Study quality assessment scores varied between 4 and 7, with a mean total score of 5.67; 38 studies (97.4%) were considered moderate quality, while one study (2.6%) was classified as high quality. The detailed characteristics and quality assessment scores are shown in **Table 1** and **Supplementary Table S2**.

### 3.2 Mental health differences of children and adolescents between one-child and multi-child families

As indicated in **Table 2** and **Supplementary Figure S1**, the pooled SMD for mental health status score was -0.02 in terms of the MSSMHS total score (95% CI: -0.09; 0.04, I^2^ = 73.2%, P = 0.47), indicating no significant difference between children from one-child and those from multi-child families. In contrast, the pooled SMD for the MHT total score was -0.13 (95% CI: -0.28; 0.01, I^2^ = 60.0%, P = 0.06), indicating a small but statistically significant difference, although not clinically meaningful. Sub-dimension analysis further revealed that significant group differences were observed in certain MHT domains scores, including Learning Anxiety (M1) [95% confidence interval (CI): -0.19; 0.00, I² = 0.0%, P = 0.04], Social Anxiety (M2) (95% CI:-0.25; 0.00, I² = 45.8%, P = 0.04), Tendency Towards Self-Blame (M4) (95% CI: -0.23; -0.07, I² = 0.0%, P < 0.01) and Allergic Tendencies (M5) (95% CI: -0.25; -0.01, I² =43.5%, P = 0.04). In all MSSMHS domains and other MHT domains, no significant group differences were found (**Table 2**).

**Table 2:** Summary of the total and MSSMHS domain scores between only child and non-only child groups.

Domain		No. of studies	Sample size		SMD	95% CI	Z	P	Heterogeneity		
			Only child	Non-only child					Q	I^2^ (%)	P value
Global mental health		39	11,889	13,795	-0.04	-0.10; 0.02	-1.23	0.22	142.08	73.3	< 0.01
MSSMHS	Total (mean)	33	10,475	12,040	-0.02	-0.09; 0.04	-0.71	0.47	119.30	73.2	<0.01
	F1	32	10,213	11,477	0.00	-0.07; 0.07	0.12	0.91	144.54	78.6	<0.01
	F2	32	10,213	11,477	-0.02	-0.07; 0.03	-0.77	0.44	75.26	58.8	< 0.01
	F3	32	10,213	11,477	0.03	-0.07; 0.12	0.47	0.64	193.76	84.0	< 0.01
	F4	32	10,213	11,477	-0.02	-0.08; 0.05	-0.46	0.65	138.51	77.6	< 0.01
	F5	32	10,213	11,477	-0.03	-0.10; 0.03	-0.94	0.34	128.17	75.8	< 0.01
	F6	32	10,213	11,477	-0.03	-0.09; 0.03	-1.07	0.28	99.13	68.7	< 0.01
	F7	32	10,213	11,477	0.00	-0.05; 0.06	0.14	0.89	80.68	61.6	< 0.01
	F8	32	10,213	11,477	-0.02	-0.07; 0.04	-0.63	0.53	82.84	62.6	< 0.01
	F9	32	10,213	11,477	0.00	-0.06; 0.05	-0.23	0.82	81.25	61.8	< 0.01
	F10	32	10,213	11,477	-0.03	-0.09; 0.03	-0.94	0.35	93.68	66.9	< 0.01
MHT	Total (mean)	6	1,414	1,755	-0.13	-0.28; 0.01	-1.85	0.06	12.50	60.0	0.03
	M1	6	1,414	1,755	-0.09	-0.19; 0.00	-2.02	0.04	4.41	0.0	0.49
	M2	6	1,414	1,755	-0.13	-0.25; 0.00	-2.03	0.04	9.22	45.8	0.10
	M3	6	1,414	1,755	-0.06	-0.21; 0.08	-0.86	0.39	13.91	64.1	0.02
	M4	6	1,414	1,755	-0.15	-0.23; -0.07	-3.57	<0.01	1.77	0.0	0.88
	M5	6	1,414	1,755	-0.13	-0.25; -0.01	-2.09	0.04	8.84	43.5	0.12
	M6	6	1,414	1,755	-0.06	-0.25; 0.12	-0.68	0.50	21.45	76.7	<0.01
	M7	6	1,414	1,755	-0.07	-0.24; 0.10	-0.84	0.40	15.38	67.5	<0.01
	M8	6	1,414	1,755	-0.10	-0.20; 0.00	-1.91	0.06	5.99	16.6	0.31

F1, Obsessive Symptoms; F2, Paranoia; F3, Hostility; F4, Interpersonal Sensitivity; F5, Depression; F6, Anxiety; F7, Academic Stress; F8, Maladjustment; F9, Emotional Instability; F10, Psychological Imbalance; M1, Learning Anxiety; M2, Social Anxiety; M3, Tendency Towards Solitude; M4, Tendency Towards Self-Blame; M5, Allergic Tendencies; M6, Physical Symptoms; M7, Tendency Towards Fear; M8, Impulsive Tendencies; MHT, Mental health test; MSSMHS, Middle School Student Mental Health Scale; SMD, Standardized mean difference.

### 3.3 Publication bias and sensitivity analyses

**Figure 2:**
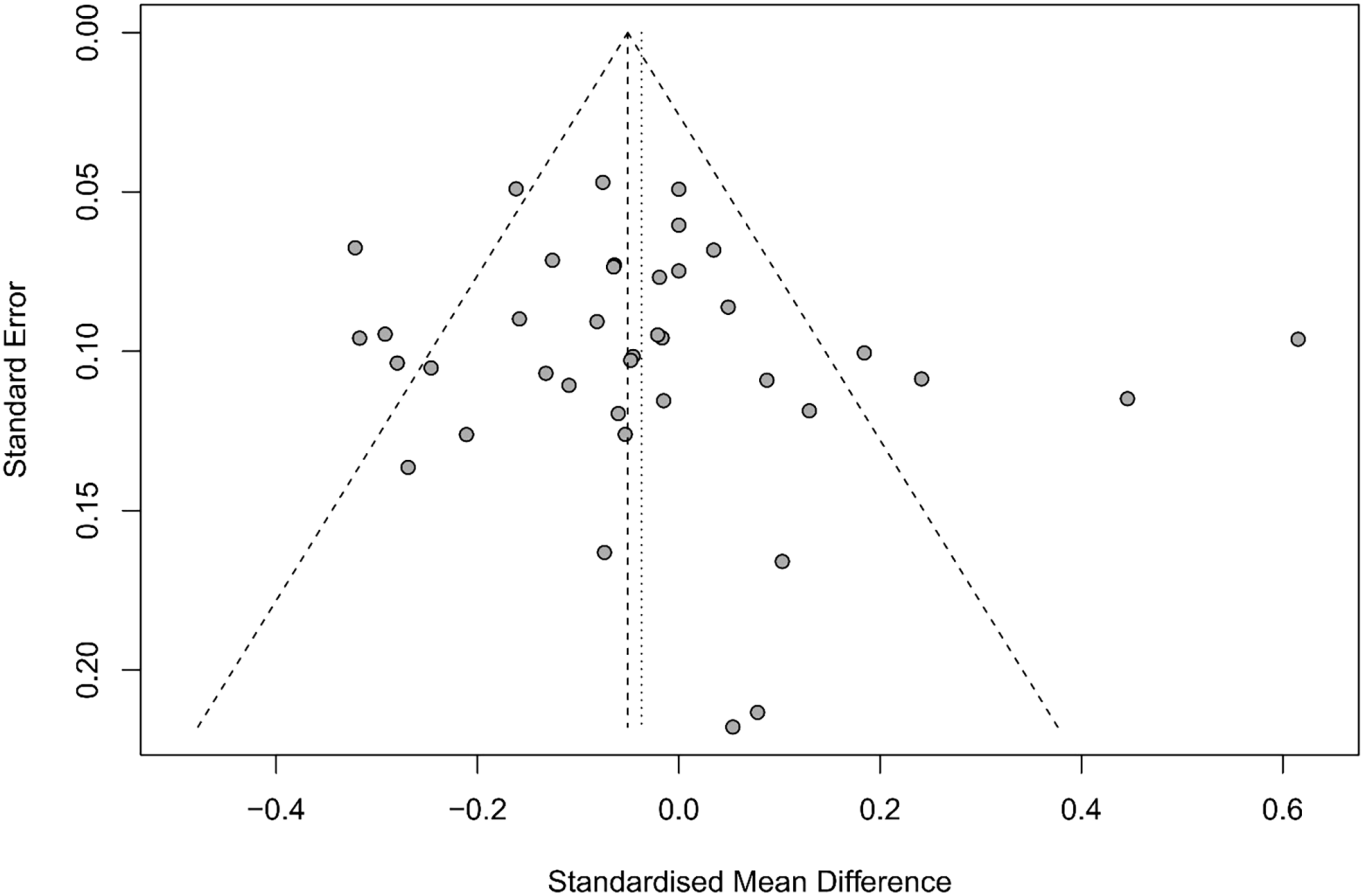
Funnel plots of publication bias of included studies.

Both Egger’s test and funnel plot analysis did not show any significant publication bias (t = 0.97, df = 37, P = 0.34; **Figure 2** and **Supplementary Figure S6**). Sensitivity analyses did not reveal any individual study that significantly altered the primary results when each was sequentially removed (**Supplementary Figure S2**).

### 3.4 Subgroup and meta-regression analyses

In the subgroup analysis, no significant moderators were identified. Differences in the mental health status between both groups were not significantly associated with population types (Q = 1.95, P = 0.38), geographical region (Q = 7.71, P = 0.05), publication year (Q = 0.01, P = 0.93) and the scales used (Q = 1.96, P = 0.16). In the meta-regression analyses, sample size (β = 0.00, P = 0.91), study quality (β = 0.07, P = 0.20), and age (β = 0.00, P = 0.89) were also not significantly associated with group differences in mental health status (**Table 3**; **Supplementary Figures S3-5**).

**Table 3:** Subgroup and meta-regression analyses of the mental health status between only child and non-only child groups.

Subgroups	Categories	No. of studies	Sample size		SMD	95% CI	I^2^ (%)	P value within subgroup	Q (P value across subgroups)
			Only child	Non-only child					
Population	Senior high	13	3,805	4,610	-0.06	-0.15; 0.02	63.0	<0.01	1.95 (0.38)
	Junior high	21	6,657	7,124	-0.06	-0.14; 0.01	64.5	<0.01	
	Both	5	1,427	2,061	0.12	-0.13; 0.37	89.1	<0.01	
Publication year	2001-2014	14	5,902	4,554	-0.04	-0.16; 0.08	82.4	<0.01	0.01 (0.93)
	2015-2023	25	5,987	9,241	-0.03	-0.10; 0.03	64.4	<0.01	
Regions	Central	7	2,588	2,631	-0.11	-0.17; -0.05	0.0	0.59	7.71 (0.05)
	Northeast	3	1,650	1,000	-0.14	-0.27; -0.02	29.5	0.24	
	Western	8	1,653	2,286	0.07	-0.06; 0.21	66.4	<0.01	
	Eastern	20	4,992	7,175	-0.04	-0.14; 0.05	80.7	<0.01	
Scales	MSSMHS	33	10,475	12,040	-0.02	-0.09; 0.04	73.2	<0.01	1.96 (0.16)
	MHT	6	1,414	1,755	-0.13	-0.28; 0.01	60.0	0.03	

Meta-regression analysis									
	Mean	No. of studies	Only-child	Non-only child	Coefficient	SE	95% CI	Z	P
Sample size	660.51	39	11,889	13,795	0.00	0.00	0.00; 0.00	-0.12	0.91
Study quality	5.67	39	11,889	13,795	0.07	0.05	-0.03; 0.17	1.27	0.20
age	15.53	6	2,280	1,975	0.00	0.03	-0.06; 0.05	-0.14	0.89

CI, Confidence interval; MHT, Mental health test; MSSMHS, Middle School Student Mental Health Scale; SE, Standard error; SMD, Standardized mean difference.

## 4 Discussion

This meta-analysis of studies from 2001 to 2022 found no significant differences in mental health status between secondary school students from one-child families and multi-child families in China. These findings appear inconsistent with those of previous reviews (26, 27) using the SCL-90 that found that the mental health status of children from one-child families was better than those from multi-child families. The difference between the findings might be attributed to the use of different rating questionnaires. Additionally, the previous studies included participants from all age groups, whereas our meta-analysis targeted only secondary school students.

The lack of significant differences between one-child and multi-child families might also be explained by the changes in societal perceptions and parenting style over time regarding children from one-child families. During the initial phases of China’s one-child policy, the restrictions on having multiple children led to public resistance towards one-child families and negative stereotypes of selfishness in those growing up in one-child families (74, 75). However, as being an only child became increasingly common in China, this might have led to a shift in public perception and a reduction in discrimination against those from one-child families (26).

The heavy academic workload and intense pressure faced by Chinese secondary school students in their studies (76), apply equally to those from one-child families and multi-child families. Due to the high rates of mental health problems among secondary school students, the mental well-being of this population has been prioritized by the Chinese government (77). Many secondary schools in China have access to well-trained mental health counselors (76), mental health courses (77) and regular mental health screenings (78). Additionally, comprehensive mental health services, including mental health assessment, public education, counseling, and intervention, have been implemented in most secondary schools nationwide (79). Those with severe mental health problems would be granted a leave of absence from school, which is considered to have therapeutic benefits (80). The absence of differences in mental health between students from one-child families and those from multi-child families in this meta-analysis may reflect the effectiveness of national policies in improving the mental health of secondary school students.

In our study, students from multi-child families had higher scores on learning anxiety, social anxiety, allergic tendencies, and self-blame tendencies compared to those from one-child families. This might indicate that students from multi-child families were more prone to experience anxiety compared to their counterparts from one-child families, which is consistent with the findings of previous research using the Generalized Anxiety Disorder Scale that secondary school students from multi-child families were more likely to experience symptoms of anxiety (81). Children in multi-child families might need to compete for parental attention and care and also face more comparison and competition with their siblings. Although having siblings might provide social interactions and support, it could also create competition, jealousy, and conflict (82, 83), all of which could exacerbate their anxiety. Moreover, sibling abuse could also lead to heightened feelings of guilt and self-blame (84).

The strengths of this meta-analysis included the focus on secondary school students alone and the inclusion of studies using scales specifically developed for secondary school students, which decreased the heterogeneity of the included studies and increased the validity of the results. However, some limitations should be noted. Junior and senior secondary school students were not differentiated in most studies, despite their different physical and psychological characteristics, and potential stressors. Furthermore, previous research found significant differences in mental health status between one-child families and multi-child families among female and rural secondary school students (85). However, the data on place of residence (rural vs. urban), and gender differences between children from one-child families and multi-child families were not recorded in most studies; therefore, the influence on the results could not be examined.

In summary, this meta-analysis found no significant difference in the mental health status between secondary school students from one-child families and multi-child families, although group differences existed in certain domains. Future research should investigate the influence of socio-demographic factors, such as gender and place of residence, on the mental health of secondary school students.

## Data Availability

The original contributions presented in the study are included in the article/**Supplementary Material**, further inquiries can be directed to the corresponding author/s
