# Supplementary material for: Mental health status of secondary school students: a meta-analysis of comparative studies between one-child and multi-child families in China
Source: Front Psychiatry. 2025 Aug 7;16:1594968. doi: 10.3389/fpsyt.2025.1594968 (PMC12368347; doi:10.3389/fpsyt.2025.1594968)

**Supplementary materials**

Table S1: Search keywords

Table S2. Quality assessment of studies included in this meta-analysis

Figure S1. Forest plots of global mental health between only child and non-only child

Figure S2. Forest plots of sensitivity analysis

Figure S3. Meta-regression analysis of sample size

Figure S4. Meta-regression analysis of quality assessment score

Figure S5. Meta-regression analysis of age

Figure S6. Egger’s test of publication bias

Table S1: Search keywords

| ("Only child" OR "single-child" OR "single child" OR "one child" OR “one-child” OR “Child, Only” OR “Only Children” OR “Children, Only”) |
| --- |
| ( “psychiatr*” OR “mental*” OR “psycholog*” OR “depress*” OR “major depress*” OR “anxie*” OR “Sleep Initiation and Maintenance Disorders” OR “insomnia” OR “sleep*” OR “posttraumatic stress disorder” OR “PTSD” OR “trauma” OR “bipolar*” OR “manic disorder” OR “schizophre*” OR “attention deficit hyperactivity disorder” OR “ADHD” OR “autism*” OR “autistic disorder” OR “obsessive compulsive disorder” OR “suicid*” OR “self-harm” OR “self-injury”) |
| (“China” OR “Chinese”) |

Table S1. Quality assessment of studies included in this meta-analysis

| No. | First Author and publication year | Item 1 | Item 2 | Item 3 | Item 4 | Item 5 | Item 6 | Item 7 | Item 8 |
| --- | --- | --- | --- | --- | --- | --- | --- | --- | --- |
| 1 | Cai (2018) | 1 | 1 | 1 | 0 | 1 | 1 | 1 | 0 |
| 2 | Chen (2014) | 1 | 1 | 1 | 0 | 1 | 1 | 1 | 0 |
| 3 | Cheng (2006) | 1 | 1 | 1 | 0 | 1 | 1 | 1 | 0 |
| 4 | Feng (2013) | 1 | 1 | 1 | 0 | 0 | 1 | 1 | 0 |
| 5 | Ge (2012) | 1 | 1 | 1 | 0 | 1 | 1 | 1 | 0 |
| 6 | Guo (2019) | 1 | 0 | 1 | 0 | 1 | 1 | 1 | 0 |
| 7 | Han (2022) | 1 | 0 | 1 | 0 | 1 | 1 | 1 | 0 |
| 8 | Huang (2017) | 1 | 1 | 1 | 1 | 1 | 1 | 1 | 0 |
| 9 | Li (2001) | 1 | 0 | 0 | 0 | 1 | 1 | 1 | 0 |
| 10 | Li (2017) | 1 | 1 | 1 | 0 | 1 | 1 | 1 | 0 |
| 11 | Li (2021) | 1 | 0 | 1 | 0 | 1 | 1 | 1 | 0 |
| 12 | Liu (2012) | 1 | 1 | 1 | 0 | 1 | 1 | 1 | 0 |
| 13 | Liu (2017) | 1 | 0 | 1 | 0 | 1 | 1 | 1 | 0 |
| 14 | Liu (2017) | 1 | 0 | 1 | 0 | 1 | 1 | 1 | 0 |
| 15 | Liu (2020) | 1 | 1 | 1 | 0 | 1 | 1 | 1 | 0 |
| 16 | Liu (2011) | 1 | 1 | 1 | 0 | 1 | 1 | 1 | 0 |
| 17 | Lu (2019) | 1 | 1 | 1 | 0 | 1 | 1 | 1 | 0 |
| 18 | Luo (2017) | 1 | 1 | 1 | 0 | 1 | 1 | 1 | 0 |
| 19 | Ma (2017) | 1 | 1 | 1 | 0 | 1 | 1 | 1 | 0 |
| 20 | Peng (2017) | 1 | 1 | 1 | 0 | 1 | 1 | 1 | 0 |
| 21 | Qiao (2016) | 1 | 1 | 1 | 0 | 1 | 1 | 1 | 0 |
| 22 | Qin (2019) | 1 | 1 | 1 | 0 | 1 | 1 | 1 | 0 |
| 23 | Shi (2010) | 1 | 1 | 1 | 0 | 1 | 1 | 1 | 0 |
| 24 | Sun (2016) | 1 | 1 | 1 | 0 | 1 | 1 | 1 | 0 |
| 25 | Tang (2015) | 1 | 1 | 0 | 0 | 1 | 1 | 1 | 0 |
| 26 | Tang (2010) | 1 | 1 | 1 | 0 | 1 | 1 | 1 | 0 |
| 27 | Tian (2011) | 1 | 1 | 1 | 0 | 1 | 1 | 1 | 0 |
| 28 | Wang (2018) | 1 | 1 | 1 | 0 | 1 | 1 | 1 | 0 |
| 29 | Wang (2022) | 1 | 1 | 1 | 0 | 1 | 1 | 1 | 0 |
| 30 | Wang (2021) | 1 | 0 | 1 | 0 | 1 | 1 | 1 | 0 |
| 31 | Xiang (2021) | 1 | 1 | 1 | 0 | 1 | 1 | 1 | 0 |
| 32 | Xiao (2016) | 1 | 1 | 1 | 0 | 1 | 1 | 1 | 0 |
| 33 | Xie (2020) | 1 | 1 | 1 | 0 | 1 | 1 | 1 | 0 |
| 34 | Zhang (2012) | 1 | 0 | 1 | 0 | 1 | 1 | 1 | 0 |
| 35 | Zhang (2021) | 1 | 0 | 0 | 0 | 1 | 1 | 1 | 0 |
| 36 | Zhao (2011) | 1 | 1 | 1 | 0 | 1 | 1 | 1 | 0 |
| 37 | Zhao (2014) | 1 | 1 | 1 | 0 | 1 | 1 | 1 | 0 |
| 38 | Zhao (2022) | 1 | 1 | 0 | 0 | 1 | 1 | 1 | 0 |
| 39 | Zhou (2014) | 1 | 1 | 1 | 0 | 1 | 1 | 1 | 0 |
| Note:  Item 1: Is the target population clearly defined?  Item 2: Is either of the following ascertainment methods used [must be one or the other]? (1) probability sampling, or (2) entire population surveyed  Item 3: Is the response rate ≥80%?  Item 4: Are non-responders clearly described?  Item 5: Is the sample representative of the target population?  Item 6: Are data collection methods standardized?  Item 7: Are validated criteria used to assess for the presence/absence of disease?  Item 8: Are the estimates of prevalence given with confidence intervals and in detail by subgroup (if applicable)? | | | | | | | | | |

Figure S1. Forest plots of global mental health between only child and non-only child


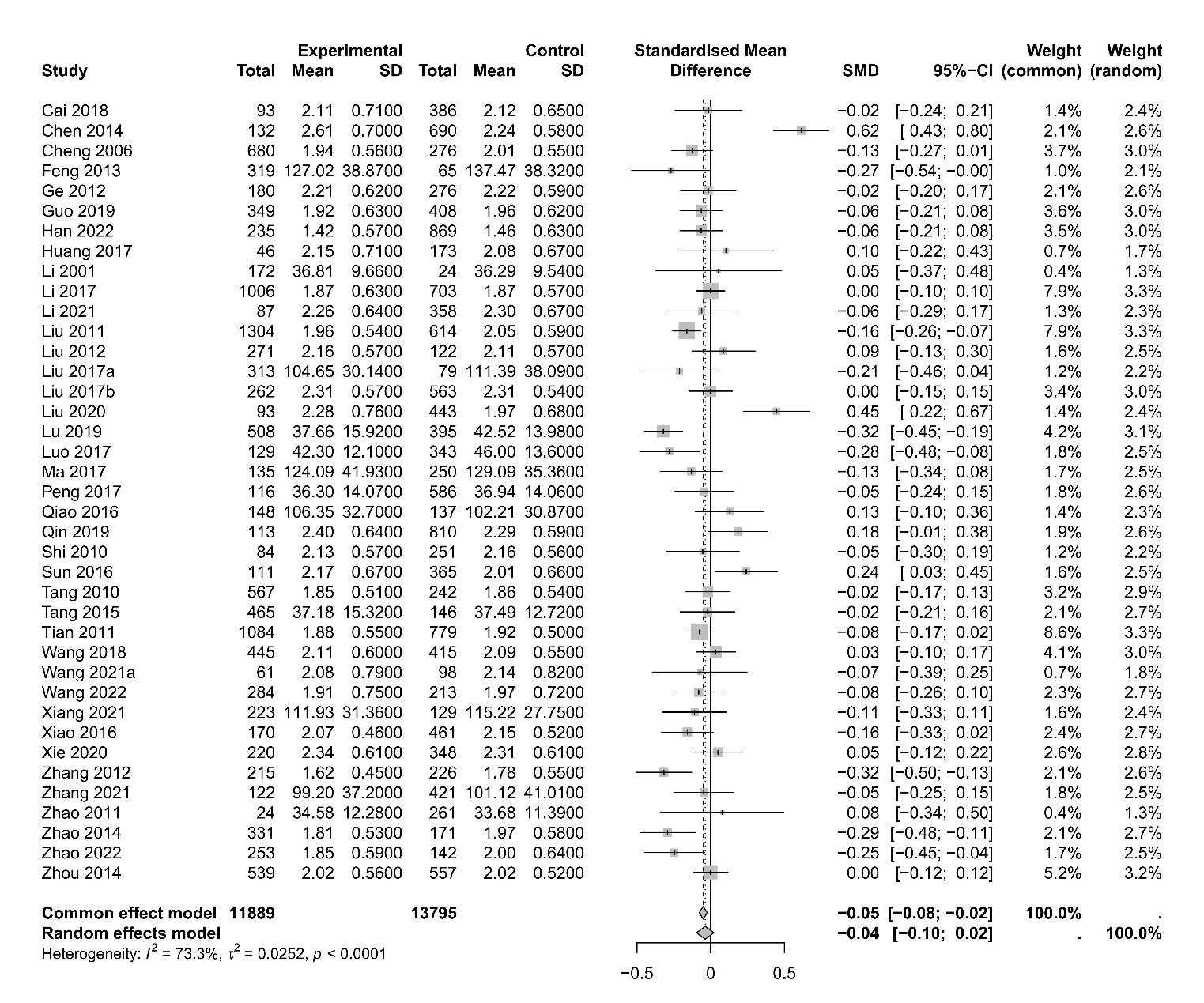


Figure S2. Forest plots of sensitivity analysis


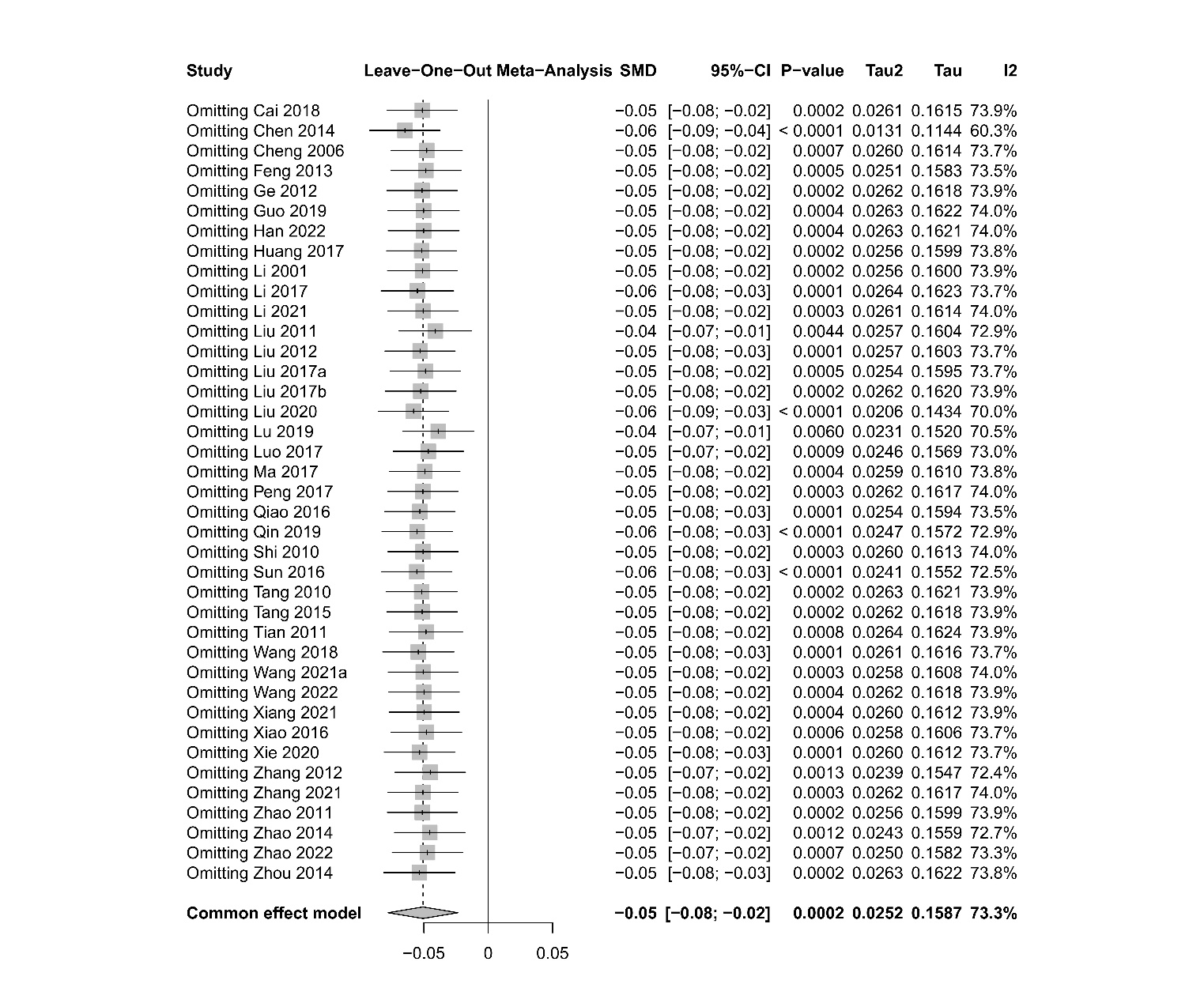


Figure S3. Meta-regression analysis of sample size


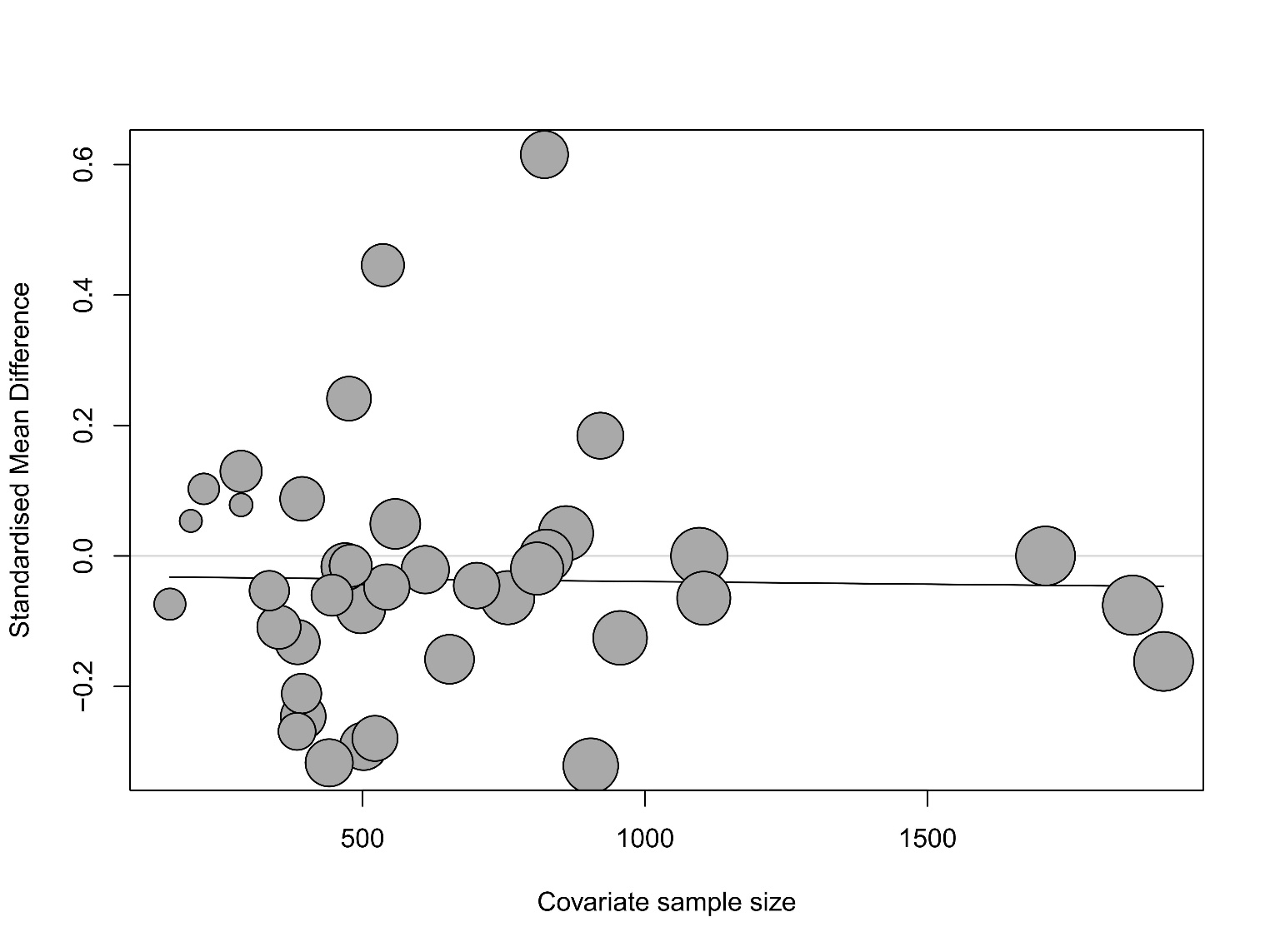


Figure S4. Meta-regression analysis of quality assessment score


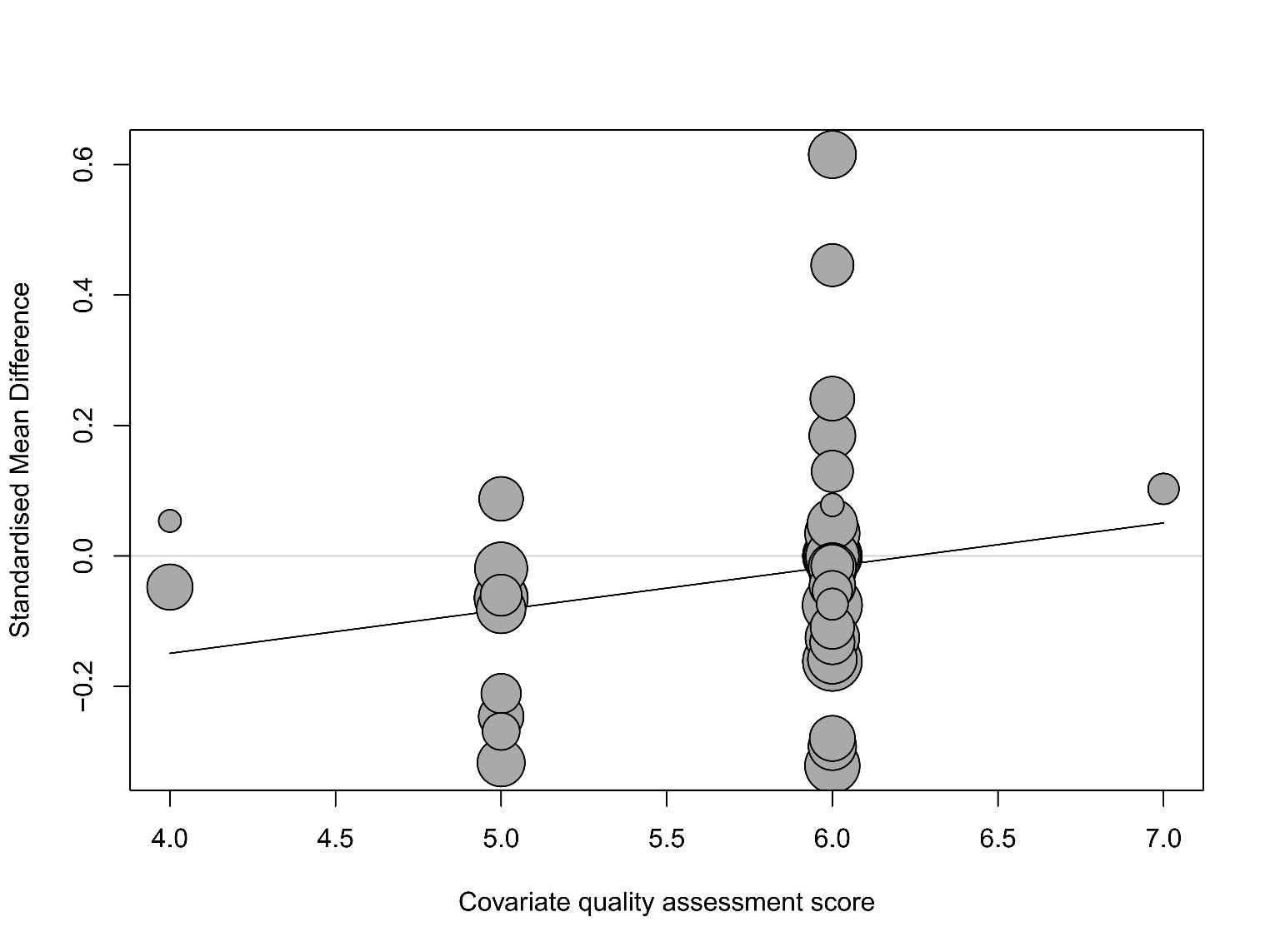


Figure S5. Meta-regression analysis of age


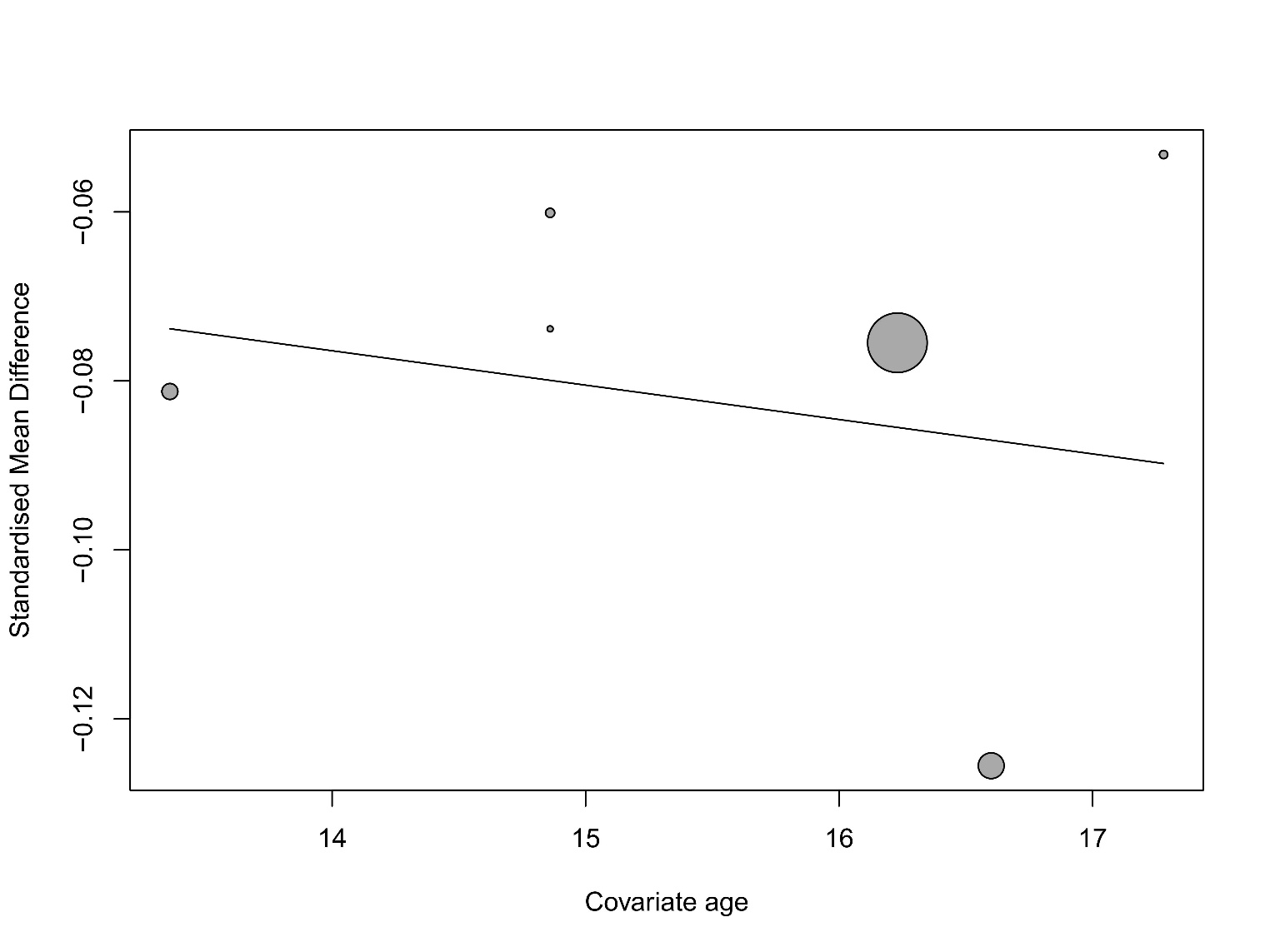


Figure S6. Egger’s test of publication bias


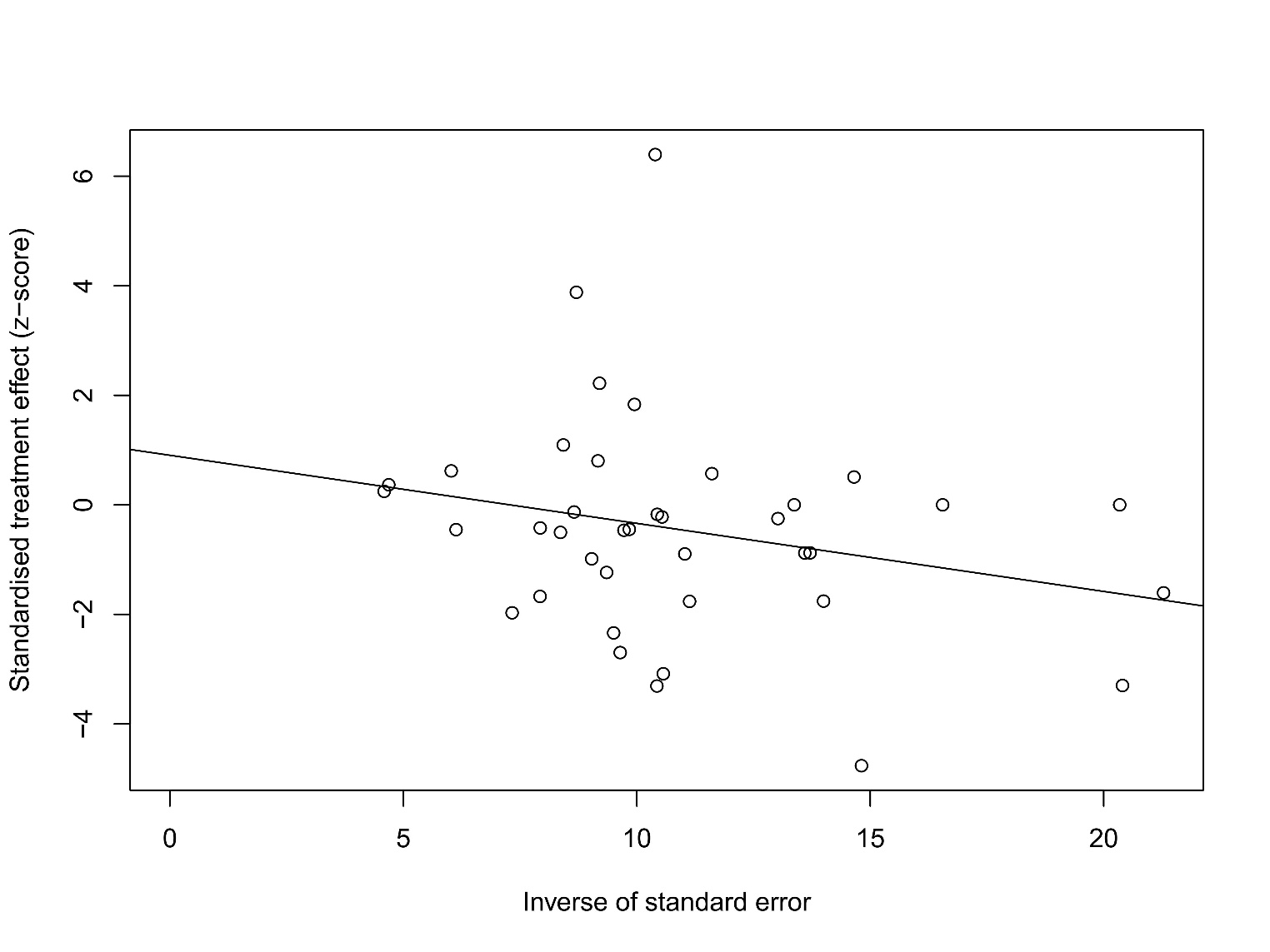

Supplement: Supplementary file 1 [file SupplementaryFile1.docx]
